# Interactions between plant endomembrane systems and the actin cytoskeleton

**DOI:** 10.3389/fpls.2015.00422

**Published:** 2015-06-09

**Authors:** Pengwei Wang, Patrick J. Hussey

**Affiliations:** School of Biological and Biomedical Science, Durham University, Durham, UK

**Keywords:** actin cytoskeleton, endomembrane system, membrane-cytoskeleton interactions, NET super-family, *Arabidopsis*

## Abstract

Membrane trafficking, organelle movement, and morphogenesis in plant cells are mainly controlled by the actin cytoskeleton. Not all proteins that regulate the cytoskeleton and membrane dynamics in animal systems have functional homologs in plants, especially for those proteins that form the bridge between the cytoskeleton and membrane; the membrane-actin adaptors. Their nature and function is only just beginning to be elucidated and this field has been greatly enhanced by the recent identification of the NETWORKED (NET) proteins, which act as membrane-actin adaptors. In this review, we will summarize the role of the actin cytoskeleton and its regulatory proteins in their interaction with endomembrane compartments and where they potentially act as platforms for cell signaling and the coordination of other subcellular events.

## Introduction

The actin cytoskeleton is involved in most aspects of plant cell development, cell morphogenesis and establishment and maintenance of cell polarity. It is a dynamic network that is responsive to extracellular and intracellular signals including mechanical stimulation (Hardham et al., 2008) and hormones (Lanza et al., 2012; Li et al., 2014). The endomembrane system is essential for intracellular protein processing, lipid modification and transport, where any dysfunction can affect plant development and signal transduction (Surpin and Raikhel, 2004; Debono et al., 2009; Ding et al., 2012; Boutté and Moreau, 2014). Each of these compartments has to maintain its structural integrity as well as their own chemical and physical properties (such as pH, redox-potential, ion composition), thereby generating a suitable environment for the function of their resident protein components (Jurgens, 2004; Martiniére et al., 2013; Schumacher, 2014).

The interaction between the actin cytoskeleton and the endomembrane system affects various aspects of plant cell function and development (Hussey et al., 2006; Sampathkumar et al., 2013), and remains an area to be more fully explored. Some of the known membrane adaptors proteins do not have homologs in plants (e.g., α-actinin and filamin; Hussey et al., 2002). Therefore, plants must have evolved their own protein complexes that localize at the cytoskeleton-membrane interface and mediate any crosstalk between compartments. In this review we focus on proteins that localize at the plant cytoskeleton-membrane interface acting as structural adaptors between the membrane and the cytoskeleton or as platforms that mediate communication between different membrane compartments (Table 1).

**TABLE 1:** **Putative actin-membrane adaptor proteins found in plants**.

**Membrane compartments**	**Proteins**	**Phenotypes/functions**	**Notes**	**Reference**
Nuclear envelope (NE)	SINEl	Positioning of nuclei in guard cells	Interact with actin through N-term; NE localization depends on SUN proteins	Zhou et al. (2014)
	Formin8	Cell division and root development	Bundle actin filament *in vitro*	Xue et al. (2011)
	Myosin VIII	N/A	Localize to the NE as tail domain deletion mutant	Golomb et al. (2008), Avisar et al. (2009)
	Myosin Xl-I	Nuclear shape and movement	Anchoring to the NE by interacting with WIT1/WIT2	Tamura et al. (2013)
	NET3A	N/A	Localize to the NE, and interact with actin through N-term	Deeks et al. (2012), Hawkins et al. (2014)
Endoplasmic reticulum (ER)	Myosins (XI-l; XI-2; XI-C; XI-K, etc.)	Root hair development and cell expansion	Affect the dynamics and structure of ER/actin. Xl-K is also enriched in ER microsome	Peremyslov et al. (2010), Ueda et al. (2010)
	NAP1	Trichome development	Form ER associated punctae	Zhang et al. (2013a)
Golgi/ER interface	SPIKE1	Cytoskeleton organization, cell morphology	Co-localize with ERES marker, affect ER morphology	Zhang et al. (2010)
	CP	Hypocotyl and root development	Localize at the *cis*-Golgi, activity negatively regulated by phosphatidie acid	Li et al. (2012), Jimenez-Lopez et al. (2014)
Vacuole	NET4A	N/A	Localized around tonoplast in root meristem cells	Deeks et al. (2012)
Plasma membrane (PM)	BRK1 and Scarl	Trichome development	Enriched in cell border and corner	Dyachok et al. (2008)
	Myosins (VIII)	N/A	N/A	Golomb et al. (2008)
	Formins (FHl, 4, 5, 8)	Root hair development (FH4, 8), pollen tube and polarized cell growth (FH1, 5).	Capable for both actin and microtubule interaction.	Cheung et al. (2010), Deeks et al. (2010), Van Gisbergen et al. (2012), Martiniére et al. (2011)
	PLD	Cytoskeleton organization	Interact with actin, microtubules and MAP65	Pleskot et al. (2010), Zhang et al. (2012)
	NET2A	N/A	May form signaling complex with protein kinase at the PM of pollen tube	Skirpan et al. (2006), Deeks et al. (2012)
ER/PM contact sites	VAP27 and NET3C	Pollen and embryo development	NET3C/VAP27 in a complex, interact with actin and microtubules respectively	Wang et al. (2014)
Plasmodesmata	Myosin VIII	N/A	N/A	Golomb et al. (2008)
	NET1A	Root development	NET super-family	Deeks et al. (2012)
Chloroplast (Cp)	CHUPl and KAC	Cp movement and anchorage	N/A	Kadota et al. (2009), Suetsugu et al. (2010)
	Myosin Xl-F	Cp and mitochondria arrangement	Localize to the Cp body and stromules	Sattarzadeh et al. (2009)

## Actin-Nuclear Membrane Interaction

The nuclear envelope (NE) is a double membrane structure that separates the chromatin and nuclear content from the cytoplasm. It breaks down during mitosis and reassembles around the newly formed sister nuclei (Graumann and Evans, 2011). The outer membrane is continuous with the ER and the inner membrane is in close association with the chromatin through the interaction of inner membrane proteins (Meier, 2007). The SUN (SAD1/UNC84) and KASH (Klarsicht/Anc/Syne-1 homology) domain protein complex is one of the best known examples for the interaction between the NE and the cytoskeleton. Such complexes are present in plants and animals and their organization is similar. The SUN proteins localize at the inner nuclear membrane and interact with KASH domain proteins at the outer membrane, which associate with the cytoskeleton (Graumann et al., 2014). The shape of the nucleus is regulated by both proteins as knock-out mutants exhibit a less elongated nuclear membrane structure (Zhou et al., 2011). SINE1 (SUN-domain Interacting NE protein 1) is the well characterized KASH-like protein in plants. It interacts with F-actin through its N-terminal sequence and also localizes to the NE in guard cells and non-differentiated root cells (Zhou et al., 2014).

Formins are conserved proteins that regulate actin dynamics by functioning at the “barbed end” of the actin filament (Blanchoin and Staiger, 2010). A study on *Arabidopsis* formin8 demonstrated that this protein induces actin bundling and a population of the endogenous protein was shown to localize to the NE (Xue et al., 2011). Moreover, Myosin XI-I is recruited by interacting with NE proteins, such as WPP domain-interacting proteins (WIPs, also known as putative plant KASH proteins; Tamura et al., 2013). It is required for maintaining the morphology and regulating the movement of the nucleus in root hair cells and in leaf mesophyll cells after dark treatment (Tamura et al., 2013). Movement of nuclei is especially important during fertilization where the vegetative nucleus moves from the pollen grain to the tip of pollen tubes. This process is known to be regulated by WIPs and myosin XI-I (Zhou and Meier, 2014).

## Actin-Endoplasmic Reticulum Interaction

The endoplasmic reticulum (ER) is involved in the synthesis, folding and quality control of membrane and luminal proteins destined for secretion, Ca^2+^ storage, lipid, and auxin biosynthesis (Vitale and Denecke, 1999; Wang et al., 2011; Kriechbaumer et al., 2012). The dynamic property of the ER is mainly regulated by the actin cytoskeleton, which is closely associated with the ER network (Boevink et al., 1998; Sparkes et al., 2011). There is some evidence that microtubules also influence the mobility of the ER, but to a lesser extent or at a much slower rate (Hamada et al., 2014).

Myosin XI isoforms (13 isoforms in *Arabidopsis*) exhibit differential effects on the inhibition of ER remodeling, and they are the most well studied proteins regulating this process. Myosin XI isoforms have an N-terminal motor domain, a regulatory neck domain and a C-terminal tail domain. Tail domain deletion mutants are commonly used to study the effect of myosin on organelle movement; these proteins could potentially be recruited to the destination membrane but they will have no actin association capacity. The over-expression of such dominant-negative mutants of myosin XI isoforms in tobacco leaf enhance the persistency of ER membrane, as well as altering ER morphology (Sparkes et al., 2009a; Griffing et al., 2014). Myosin XI-K is also found to be enriched in the ER microsomal fractions and localizes to membrane structures along the F-actin (Peremyslov et al., 2012). Its deficiency affects the organization of the ER network and the associated actin filaments (Ueda et al., 2010). Due to the diversity of myosin tail domains, each may localize to multiple membrane compartments and regulate the movement of organelles other than the ER including peroxisomes and mitochondria (Hashimoto et al., 2005; Avisar et al., 2008a; Peremyslov et al., 2008, 2010; Sparkes et al., 2008; Sattarzadeh et al., 2013). However, it is not clear whether the inhibition of their mobility is directly related to less actin cytoskeleton association or a global effect of reduced cytoplasmic or ER streaming.

In mammals, disturbing any ER-microtubule association or removing the microtubule network alters the ER morphology (Vedrenne et al., 2005; Vedrenne and Hauri, 2006). In most plant cells, treatment with a drug that has the net effect of F-actin depolymerization (Latrunculin B) inhibits the movement and remodeling of the ER, but its effect on ER morphology is less significant (Sparkes et al., 2009a). Some *in vitro* studies on ER microsomes from BY2 cells claim that the formation of the ER network does not require a functional cytoskeleton, as isolated ER microsomes are able to assemble into a polygonal structure. However, this process is significantly inhibited by depletion or over-expression of dominant-negative myosins (Yokota et al., 2011).

## Actin-ER/Golgi Interface Interaction

The Golgi receives secretory vesicles containing proteins and other substances from the ER, to its *cis*-face and sends the sorted materials to their destinations (Hawes, 2005). Golgi bodies are physically connected to the ER surface in higher plants (Sparkes et al., 2009b); they interact with the ER through a proteinaceous bridge, which contains COPII proteins required for ER exit site (ERES) formation (Hanton et al., 2009). Interactions between microtubules and ERES components have been described in animals and these involve direct interaction with Sec23p (coat protein of COPII vesicles) and the dynactin complex (Watson et al., 2005).

In plants, the actin cytoskeleton substitutes for microtubules as the major cytoskeletal component involved in membrane trafficking, so it is reasonable to speculate that an analogous phenomenon exists where actin associated protein complexes are involved in bridging the links. Indeed, recent studies revealed that the ER network is likely to be one of the main reservoirs for the Scar/Wave signaling complex, and the ARP2/3 complex (Zhang et al., 2013a,b), whose activation promotes actin filament branching and polymerization (Deeks and Hussey, 2005; Uhrig et al., 2007). SPIKE1, a guanine nucleotide exchange factor that acts up-stream of the Scar/Wave complex, is found to be ERES localized. Its depletion changes ER morphology as well as the localization of ERES components (Zhang et al., 2010). In addition, the barbed end actin binding protein, capping protein (CP), has recently been identified as associating with a *cis*-Golgi marker (Jimenez-Lopez et al., 2014), which raises the possibility that plant ERES are also sites where actin dynamics are regulated.

In animal cells, the interaction between the microtubule cytoskeleton and ERES is required for the movement and formation of the ER-Golgi intermediate compartment (ERGIC), which is essential for the secretory pathway (Appenzeller-Herzog and Hauri, 2006). So, what is the function of actin regulatory proteins (e.g., Scar complexes and CP) that localize to the ER/Golgi in plants? In ARP2/3 mutants, Golgi bodies are found to be trapped within dense patches of actin filaments within the distorted trichomes; this phenomenon could possibly result in an altered Golgi mobility (Mathur et al., 2003). Moreover, it is known that plants do not have ERGIC like structures, and it has been debated for decades that Golgi and ER are connected forming a “secretory unit” (Robinson et al., 2015). Disrupting the cytoskeleton has no obvious effect on ER-Golgi trafficking in tobacco cells (Brandizzi et al., 2002; Saint-Jore et al., 2002). Therefore, the function of these actin regulatory proteins in ER-Golgi “vesicle trafficking” is an area open to investigation.

## Actin-Vacuole Interaction

Plant vacuoles are sub-divided into two groups: the protein storage vacuoles (PSVs) and the lytic vacuoles (LVs; Hunter et al., 2007; Olbrich et al., 2007). The transition between the two types of vacuole has been observed in root meristematic cells (PSV to LV) and cells at the early embryo stage (LV to PSV; Feeney et al., 2013). The actin network is found at the tonoplast surface where it regulates vacuole structure and dynamics (Higaki et al., 2006; Sheahan et al., 2007). It is expected to play a major role during the process of vacuole transition, where dramatic tonoplast membrane re-organization has been observed. One hypothesis that has been suggested for the formation of the central LV is by the fusion of multiple small PSVs during development. Actin dynamics regulated by actin associated proteins may provide the force that brings PSVs close together, allowing the docking and fusion process to take place. Such processes have been reported in guard cells during stomatal opening (Li et al., 2013), and perhaps this phenomenon could be extrapolated to include other cell types. In addition, actin is also essential for membrane trafficking between the vacuole and the Golgi compartment (Kim et al., 2005).

## Actin-Plasma Membrane Interaction

The plasma membrane (PM) is a main site of association for actin regulatory proteins including subunits of the Scar/Wave complex (Dyachok et al., 2008), myosins (Golomb et al., 2008), profilin (Sun et al., 2013), and formins. Multiple plant formin homologs have been found at the PM of various cell types suggesting an important role in plant cell development and morphogenesis (Cheung and Wu, 2004; Favery et al., 2004; Deeks et al., 2005; Cheung et al., 2010; Martiniére et al., 2011). They can be recruited to the PM through direct transmembrane domain insertion (e.g., AtFH1), interacting with phospholipids (class II formins) or possibly through the indirect association with other proteins (Van Gisbergen et al., 2012; Cvrckova, 2013). Some formins have been found to have unique microtubule binding regions and are therefore capable of interacting with both actin and microtubule filaments (Deeks et al., 2010; Li et al., 2010; Zhang et al., 2011). Another example of an actin/microtubule dual associating protein is Phospholipase D (PLD), which regulates cell signaling by converting structural phospholipids to phosphatidic acid (Pleskot et al., 2010, 2013). Furthermore one study has demonstrated that PLD at the PM can bind the well-known microtubule interacting protein MAP65 in response to salt stress (Zhang et al., 2012). The PM-actin interaction is also essential for the development of stomata, which requires an asymmetrical division of subsidiary cells. Proteins from the SCAR/WAVE complex, as well as the receptor-like kinases PAN1 and PAN2, localize to the PM in a polarized pattern. This complex activates ARP2/3 dependent actin polymerization and drives nuclear movement and cell division (Facette et al., 2015).

## Actin-PM/ER Contact Sites and Plasmodesmata Interaction

Plasmodesmata (PD) are plant specific channels that traverse the cell walls between cells and where the cytoskeleton, PM and ER converge (Fitzgibbon et al., 2010). They are required for cell-to-cell communication and transport, such as the movement of viral proteins during viral infection (Avisar et al., 2008b; Tilsner et al., 2013; Amari et al., 2014). Actin filaments and certain myosins have been demonstrated previously to associate with PD. For example, myosin VIII localizes to PD (Radford and White, 1998; Golomb et al., 2008) and some myosin XI isoforms are recruited to the cell plate during cytokinesis where most primary PDs are formed (Reichelt et al., 1999; Yokota et al., 2009). As well as at PD, the ER and PM join at, what are known as, ER/PM contact sites (EPCS). They are found predominantly at the cell cortex (especially in epidermal cells), but they are also likely to be associated with the PD at cell junctions. The NET3C and VAP27 proteins, which interact with the actin and microtubule cytoskeletons respectively, are required for the formation of EPCS in plants (Wang et al., 2014). The function of the cytoskeleton-EPCS interaction in plants is not clear, one hypothesis is that such an interaction is important for cargo exchange during endocytic and exocytic trafficking (Pena and Heinlein, 2013).

## Actin-Plastid Interaction

Strictly speaking, chloroplasts are not classified as part of the endomembrane system as they are endosymbionts. But their movement is actin dependent and this movement can be light responsive (Higa et al., 2014), which presents a unique aspect of actin-membrane interaction. For example, the CHloroplast Unusual Positioning 1 (CHUP1) protein localizes to the outer membrane of chloroplasts and is bound to short actin filaments (Kadota et al., 2009). It also interacts with two kinesin-like proteins (KAC1 and KAC2), which mediate this actin-based movement (Suetsugu et al., 2010). Not surprisingly, over-expression of some of the myosin XI tail domain truncations (e.g., myosin XI-F that localize to the chloroplast body and stromules) also has an effect on chloroplast movement (Sattarzadeh et al., 2009).

## The NET Super-Family and Conclusion

**FIGURE 1:**
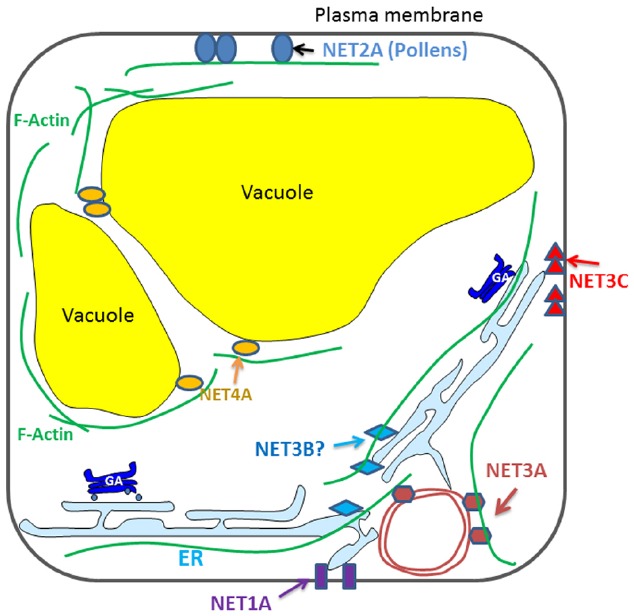
**Diagrammatic illustration of the known interactions between NET proteins and plant endomembrane system**.

Recently, the NET super-family of actin binding proteins has been identified. They interact directly with F-actin through a conserved N-terminal domain, and are recruited to different membrane compartments through a highly variable C-terminal sequence (Figure 1). A few members of the NET family have been characterized and each of them localize to distinct membrane compartments: the NE, NET3A; EPCS, NET3C; and the tonoplast, NET4A (Deeks et al., 2012; Wang et al., 2014). The mechanism of action of these proteins is only just starting to be evaluated and now represents a key area for developing our understanding of plant cytoskeleton-membrane interactions.

NET1A, the representative member of the NET1 subfamily, is a novel type of PD associated actin-membrane adaptor. Its knock out (along with NET1B) affects root development in that a long root phenotype is observed. However, the mechanism that causes this defect is unclear but it is suggested that the phenotype may be the result of aberrant transport of as yet unknown substances between cells. NET2A is pollen specific and it forms PM associated punctae that co-align with the actin cytoskeleton in pollen tubes (Deeks et al., 2012). The petunia homolog of NET2A forms a complex with PM-integrated protein kinase (PRK1) and oxysterol-binding proteins (Skirpan et al., 2006), which could indicate that this complex forms a platform for signaling between the interior and exterior of the cell. Their potential influence on male gametophyte development and pollen viability is an interesting topic for further study.

NET3A localizes to the NE as well as to actin filaments within the cytoplasm. Perhaps it could form complexes with some of the known NE localized cytoskeleton interacting proteins (e.g., myosins and WIPs), assisting in the regulation of plant nuclear structure and function. NET3B, the remaining uncharacterized member of the NET3 family, expresses strongly in pollen and vascular tissue, where NET3C is also present. The NET3B/3C double mutant exhibits defects in pollen development suggesting that there is likely to be some functional redundancy between these two proteins (Wang et al., 2014).

NET4A localizes to the tonoplast, where it forms a characteristic NET protein “beads-on-a-string” localization pattern, the beads depicting potential membrane contact sites and the string as actin filaments (Deeks et al., 2012). In contrast to other vacuole membrane intrinsic proteins, the localization of NET4A does not show any obvious alteration in response to auxin stimuli (Lofke et al., 2015). NET4A is the first protein known to bind actin filaments at the tonoplast in plants. Two NET4 homologs are found in *Arabidopsis*, both of which interact with actin filaments but they have slightly different expression profiles. Based on their localization it is possible that NET4 is involved in maintaining tonoplast structure (Hawkins et al., 2014).

So far, our knowledge of the NET family is limited. However, the studies to date would indicate that at least a few members of the family are essential in establishing and maintaining links with different membrane systems. Their mode of action is unknown and future work will revolve around understanding their function in plant cell morphogenesis. The association of the actin cytoskeleton with membrane compartments is a general phenomenon in plant cells. Adaptor proteins at the site of the actin and membrane interface are essential, not only for regulating organelle dynamics and movement, but also for providing the structural integrity and specificity for various membrane organization/fusion events during plant development. Future studies will involve identifying the components of the NET-adaptor complexes, and their mechanism of action in different subcellular events.

### Conflict of Interest Statement

The authors declare that the research was conducted in the absence of any commercial or financial relationships that could be construed as a potential conflict of interest.
